# T-cell immune status in patients with acute exacerbation of chronic obstructive pulmonary disease: a case-control study

**DOI:** 10.3389/fmed.2025.1433844

**Published:** 2025-01-24

**Authors:** Xiao-feng Xiong, Min Zhu, Hong-xia Wu, Zuo-hong Wu, Li-li Fan, De-yun Cheng

**Affiliations:** ^1^Department of Respiratory and Critical Care Medicine, West China Hospital, Sichuan University, Chengdu, China; ^2^Laboratory of Pulmonary Immunology and Inflammation, Frontiers Science Center for Disease-Related Molecular Network, Sichuan University, Chengdu, China

**Keywords:** chronic obstructive pulmonary disease, acute exacerbation, T cells, immunity, multi-color flow cytometry

## Abstract

**Introduction:**

Immune inflammatory response plays an important role in chronic obstructive pulmonary disease (COPD). However, the cellular immune status of patients with COPD at different phases is unclear. Herein, we aim to investigate the distribution and functional status of T cell subsets in different phases of COPD (acute exacerbation of COPD [AECOPD] and stable COPD [SCOPD]).

**Methods:**

This is an observational case-control study undertaken in West China Hospital. The distribution of T cell subsets in peripheral blood of AECOPD, SCOPD, and healthy controls (HCs) was measured using multi-color flow cytometry, and the functional status was analyzed by additional staining of activation markers.

**Results:**

A total of 43 HCs, 43 SCOPD patients, and 64 AECOPD patients were evaluated. The total number and percentage of lymphocytes and the CD4+/CD8+ T cells ratio were significantly lower in AECOPD patients when compared to HCs. HLA-DR expression in CD3+, CD4+, CD8+, CD8+ TCR aβ, and CD4+ TCR aβ T cells was upregulated in the AECOPD group. Similarly, the expressions of HLA-DR, CD57, and PD-1 were higher in T cell subsets in the AECOPD group. Compared with the SCOPD and HC groups, the AECOPD had a significantly lower proportion of CD4+CD27+CD28+ T cells, but opposite results were found for CD4+CD27-CD28- T cells. In addition, the proportion of CD4+CD39+ T cells and CD4+CD25+FoxP3+ T cells was significantly higher in the AECOPD and SCOPD groups when compared to the HC group (*P* < 0.05).

**Discussion:**

The distribution of nearly half the T cell subsets in AECOPD patients was significantly different from that in SCOPD patients and HCs. AECOPD patients may have cellular immune suppression, immune dysfunction, abnormal activation, and higher senescence depletion of T cells.

## 1 Introduction

Chronic obstructive pulmonary disease (COPD) is a leading cause of morbidity and mortality worldwide (1). COPD is prevalent in 13.7% of Chinese adults aged over 40 years (2), and more than 5.4 million people are projected to die from COPD and related diseases by 2060 (3, 4). COPD manifests in two distinct phases: stable COPD (SCOPD) and acute exacerbation of COPD (AECOPD). The latter can cause a rapid decline in lung function and severely impair patients’ quality of life (1).

The inflammatory response associated with COPD exhibits notable heterogeneity. This response is characterized by aberrant activation of the innate immune system, predominantly mediated by neutrophils and macrophages in pulmonary regions, coupled with systemic inflammation. Furthermore, the engagement of T lymphocytes in the adaptive immune response contributes to the chronicity and exacerbation of inflammation, which furthers the development of emphysema and culminates in airway remodeling (5). The immune-mediated inflammatory response may be pivotal in the pathogenesis and progression of COPD. However, there is ongoing debate regarding whether disparities exist in the distribution of T-cell subsets in the different phases of COPD.

The pathogenesis of COPD has yet to be fully elucidated, but the immune inflammatory response is known to play an important role in its occurrence and development. Autoimmune abnormalities have recently been found to promote the development of COPD, providing a new perspective for understanding the pathogenesis of this disease (6–10). Current clinical studies predominantly limit their scope to a primary analysis of peripheral blood T lymphocytes, including CD4+ and CD8+ cells, without an exhaustive and detailed examination of more nuanced T-cell subsets. To date, no clinical research has comprehensively observed the distribution and functional status of T-cell subsets in the peripheral blood at different phases of COPD.

Thus, this study aimed to investigate the distribution and functional status of T-cell subsets in different phases of COPD to provide a clinical and theoretical basis for understanding the immunological mechanism of COPD.

## 2 Materials and methods

### 2.1 Study design and subjects

This observational case-control study was conducted in accordance with the Declaration of Helsinki and was approved by the institutional ethics committees of West China Hospital of Sichuan University (identification no. 2018 [283]). The study was registered in the China Clinical Trials Registry on 19 September 2018 (ChiCTR1800018452). Written informed consent was obtained from all participants.

**FIGURE 1:**
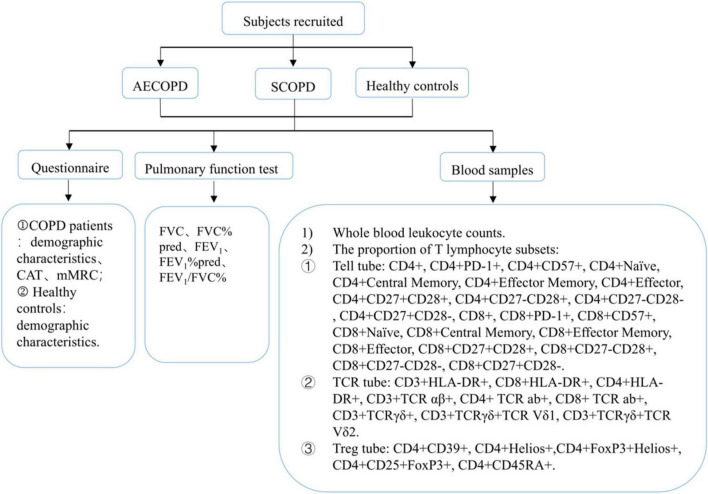
Flow diagram of the study.

All COPD patients were diagnosed according to the 2017 global initiative for chronic obstructive lung disease (GOLD) guidelines (11) and were recruited from the West China Hospital between September 2018 and October 2019. Recruitment of SCOPD patients was conducted through the hospital’s outpatient clinic, while AECOPD patients were recruited from those who visited the emergency room or were hospitalized due to symptom exacerbation. Healthy controls (HCs), who underwent physical examinations at the same hospital, were matched for age and sex with the patients. Both HCs and COPD patients were recruited according to the key inclusion and exclusion criteria (Table 1). Subject demographic and clinical characteristics, including age, sex, body mass index, smoking history, and comorbidities, were recorded. Pulmonary function tests were performed in all HCs, SCOPD patients, and AECOPD patients under permissible conditions. COPD symptoms were evaluated according to the COPD assessment test (CAT), and the modified British Medical Research Council (mMRC) dyspnea scale (12) and AECOPD patients were assessed within 48 h after admission. AECOPD patients received standardized treatment regimens, including (but not limited to) antibiotics, systemic corticosteroids, short-acting inhaled β2 agonists, short-acting anticholinergics, and oxygen therapy. The patient inclusion flowchart is shown in Figure 1.

**TABLE 1:** Inclusion and exclusion criteria of study.

Inclusion criteria	Exclusion criteria
• Age ≥ 40 years;	(1) Had a history of systemic corticosteroid therapy (prednisone > 0.5 mg/kg or equivalent doses) within 1 month;
• SCOPD is defined as respiratory symptoms were stable or mild, and the condition was stable for 3 months.	(2) Had a history of diseases that affect immune cells, such as connective tissue diseases, immunological diseases, hematological diseases, hepar and renal failure;
• AECOPD is defined as an acute worsening of respiratory symptoms that results in additional therapy.	(3) Had a history of asthma, allergic disease, or other clinically significant lung diseases;
• HCs: COPD-free individuals with normal pulmonary function.	(4) Had a history of heart failure, arrhythmia, mental disorders, or any malignancy.

AECOPD, acute exacerbation of chronic obstructive pulmonary disease; SCOPD, stable chronic obstructive pulmonary disease; HCs, healthy controls.

### 2.2 Blood collection and flow cytometry

Blood samples were collected within 3 days of symptom exacerbation and before the patients received systemic corticosteroid therapy (prednisone > 0.5 mg/kg or equivalent doses). Routine blood and T-cell subset tests were conducted within 24 h. The routine blood tests were completed by the Clinical Laboratory Department of West China Hospital of Sichuan University, and T-cell subsets were analyzed by flow cytometry.

For flow cytometry, flow cytometric fluorescent anti-human monoclonal cell surface antibody (dry powder) tubes (DuraClone IM panels: T cell, TCR, and Treg subsets) were purchased from Beckman Coulter (Brea, CA, USA). All operations were performed according to the product instructions. The fluorochrome-conjugated antibody, schemes of fluorochrome channel, and compensation controls (each of a single color) are described in Supplementary Table 1. All samples were analyzed with a 13-color CytoFlex flow cytometer (Beckman Coulter, Brea, CA, USA) after daily calibration with Flow-Set Pro Beads (Beckman Coulter, Brea, CA, USA).

### 2.3 Strategies for T-cell subset gating

The classification of T-cell subsets is shown in Supplementary Figure 1, and strategies for T-cell subset gating are shown in Supplementary Figures 2–4. We used a two-parameter scatter plot composed of CD45 and the side scatter area to gate the lymphocytes. The CD3+ cell population was defined as the total T-cell population and categorized into seven populations according to human leukocyte antigen-DR (HLA-DR), CD57, programmed death receptor 1 (PD-1), TCR, CD8, and CD4 expression as follows: (1) HLA-DR+ T cells, (2) CD57+ T cells, and (3) PD-1+ T cells. (4) TCR aβ+ T cells, (5) TCR γδ+ T cells, (6) CD8+ T cells, and (7) CD4+ T cells. CD8+ T cells were divided into 4 subtypes: (1) CD8+CD57+ T cells, (2) CD8+PD-1+ T cells, (3) costimulatory molecules (CD8+CD27+CD28+ T cells, CD8+CD27+CD28- T cells, CD8+CD27-CD28+ T cells, and CD8+CD27-CD28- T cells), and (4) CD45RA- and CCR7-expressing T cells (CD8+ effector T cells, CD8+ central memory T cells, CD8+ naïve T cells, and CD8+ effector memory T cells). Finally, CD4+ T cells were divided into the following 5 subtypes: (1) CD4+CD57+ T cells, (2) CD4+PD-1+ T cells, (3) costimulatory molecules (CD4+CD27+CD28+ T cells, CD4+CD27+CD28- T cells, CD4+CD27-CD28+ T cells, and CD4+CD27-CD28- T cells), (4) antigen responses (CD4+ effector T cells, CD4+ central memory T cells, CD4+ naïve T cells, and CD4+ effector memory T cells), and (5) regulatory T cells (CD4+CD39+ T cells, CD4+CD25+Forkhead box protein 3 (FoxP3)+ T cells, CD4+CD45RA+ T cells, CD4+Helios+ T cells, and CD4+FoxP3+Helios+ T cells).

### 2.4 Statistical analysis

Normally distributed data are described as the means ± standard deviations (SDs), whereas nonnormally distributed data are reported as medians (interquartile ranges, IQRs) unless otherwise indicated. Continuous parametric data, including CAT scores, WBC counts, pulmonary function test results indices, and T-cell subset percentages, were analyzed using Student’s *t*-test or one-way analysis of variance (ANOVA), and continuous nonparametric data were analyzed using the Mann–Whitney U or Kruskal–Wallis test. Correlation analyses between the proportion of T-cell subsets and symptom score and lung function were performed by using the Pearson or Spearman correlation test. All statistical analyses were performed using SPSS 24.0 (IBM, Armonk, NY, USA), and a *P*-value < 0.05 was considered to indicate statistical significance.

## 3 Results

### 3.1 Subject baseline characteristics

A total of 43 HCs, 43 SCOPD patients, and 64 AECOPD patients were recruited. The clinicodemographic characteristics are summarized in Table 2 and Supplementary Table 2. Compared with the SCOPD group, the AECOPD group had worse symptom control and pulmonary function.

**TABLE 2:** Demographic and clinical characteristics of subjects at baseline.

	Healthy control	SCOPD	AECOPD	*P*-value
Patients (*n*)	43	43	64	
Age, years	63.07 ± 7.87	64.12 ± 9.13	66.78 ± 7.43	0.732
Male, *n* (%)	30 (69.8)	32 (74.4)	49 (76.6)	0.051
Smoking history				0.094
Current smoker, *n* (%)	14 (32.5)	19 (44.1)	26 (40.6)	
Ever smoker, *n* (%)	10 (23.3)	14 (32.5)	19 (29.7)	
Never smoker, *n* (%)	19 (44.2)	10 (23.3)	19 (29.7)	
Smoking index^¥^	0 (0.350)	740 (100.960)	525 (0.900)	<0.0001
BMI (kg/m^2^)	22.99 ± 2.66	22.59 ± 3.08	20.04 ± 4.00	0.357
FEV_1_(L)	2.36 ± 0.52	1.57 ± 1.46^#^	1.01 ± 0.32*	<0.001
FEV_1_% pred	98.37 ± 14.55	52.49 ± 21.90^#^	45.9 ± 15.27*	<0.001
FVC(L)	2.98 ± 0.68	2.60 ± 0.84^#^	1.90 ± 0.49*^Δ^	0.001
FEV_1_/FVC (%)	78.00 ± 5.58	50.34 ± 11.61^#^	55.64 ± 19.93*	<0.001
Leukocyte count, 10^9^/L	6.91 ± 3.01	5.75 ± 1.69	8.53 ± 3.6*^Δ^	<0.001
Neutrophil count, 10^9^/L	3.44 ± 1.30	3.46 ± 1.27	6.60 ± 3.35*^Δ^	<0.001
Lymphocyte count, 10^9^/L	1.65 ± 0.59	1.69 ± 0.54	1.21 ± 0.63*^Δ^	<0.001
Monocyte count, 10^9^/L	0.37 ± 0.18	0.40 ± 0.16	0.56 ± 0.28*^Δ^	<0.001
Eosinophil count, 10^9^/L	0.11 ± 0.08	0.15 ± 0.12	0.12 ± 0.16	0.264
Neutrophil, %	60.43 ± 8.94	59.37 ± 7.72	72.79 ± 15.84*^Δ^	<0.001
Lymphocyte, %	29.72 ± 9.49	30.29 ± 7.88	15.93 ± 8.54*^Δ^	<0.001
Monocyte, %	6.40 ± 1.82	7.11 ± 1.68	6.92 ± 2.79	0.315
Eosinophil, %	1.85 ± 1.13	2.62 ± 2.12^#^	1.62 ± 1.86^Δ^	0.016

Data presented as mean ± SD unless specified.

^¥^median (interquartile range). Smoking index, the number of cigarettes per day multiplied by the number of smoking years.

*AECOPD vs. Healthy control, *p* < 0.05,

^Δ^AECOPD vs. SCOPD, *p* < 0.05,

^#^SCOPD vs. Healthy control, *p* < 0.05. AECOPD, Acute exacerbation of chronic obstructive pulmonary disease; SCOPD, Stable chronic obstructive pulmonary disease; BMI, body mass index; FEV_1_, forced expiratory volume in one second, FVC, forced vital capacity; mMRC, modified medical research council dyspnea scale; CAT, COPD assessment test.

### 3.2 Distribution of the general T-cell subsets

**FIGURE 2:**
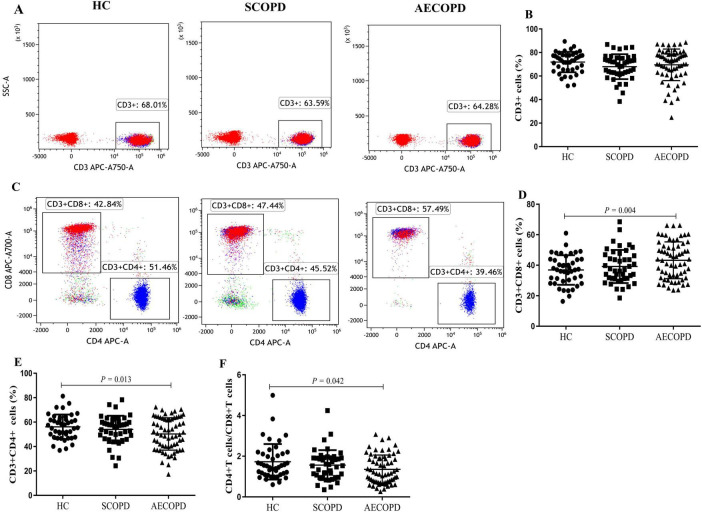
Parental proportions of general T cell subsets in peripheral blood among three groups. CD3+ T cells **(A,B)**, CD8+ T cells **(C,D)**, CD4+ T cells **(C, E)**, CD4+ /CD8+ T cells **(F)**. Data are expressed as mean number of each group (mean ± SD).

The distributions of the T-cell subsets detected for CD3+ T cells and their two main subsets (CD8+ and CD4+ T cells) are detailed in Table 3 and Figure 1. There was no significant difference in the proportion of CD3+ T cells among the three groups (Figures 2A, B). The proportion of CD8+ T cells was significantly higher in the AECOPD group than in the HC (*P* = 0.004, Figure 2C, D). In contrast, the proportion of CD3+CD4+ T cells and the CD4+/CD8+ T-cell ratio were significantly lower in the AECOPD group than in the HC group (*P* = 0.013 and *P* = 0.042, Figures 2E, F). Compared with the HCs, the SCOPD patients had a higher proportion of CD3+CD8+ T cells and a lower proportion of CD3+CD4+ T cells, but the difference was not statistically significant.

**TABLE 3:** The proportion of T cell subsets in peripheral blood of subjects.

T cell subsets	Healthy control	SCOPD	AECOPD	*P*-value
CD3+ (%)	71.87 ± 8.67	67.98 ± 10.58	69.55 ± 13.44	0.286
CD3+CD8+ (%)	36.88 ± 9.69	39.15 ± 10.86	43.21 ± 12.06*	0.013
CD3+CD4+ (%)	56.16 ± 10.03	54.04 ± 11.13	50.33 ± 13.25*	0.038
CD4+/ CD8+	1.73 ± 0.87	1.56 ± 0.74	1.35 ± 0.71*	0.045
CD3+HLA-DR+	39.10 ± 13.98	44.97 ± 12.39	48.83 ± 17.26*	0.006
CD8+HLA-DR+	58.92 ± 17.17	64.81 ± 13.04	68.95 ± 15.63*	0.005
CD4+HLA-DR+	24.24 ± 9.70	25.31 ± 10.45	29.23 ± 15.43*	0.099
CD8+TCR aβ+ HLA-DR+	58.40 ± 17.55	63.84 ± 13.22	68.59 ± 16.05*	0.005
CD4+TCR aβ+ HLA-DR+	24.01 ± 9.70	25.10 ± 10.47	29.03 ± 15.43*	0.097
CD3+TCR aβ+	92.43 ± 6.43	92.61 ± 5.74	93.88 ± 5.83	0.388
CD8+TCR aβ+	38.97 ± 12.52	39.36 ± 11.78	30.95 ± 13.53	0.692
CD4+TCR aβ+	58.66 ± 12.63	57.86 ± 11.72	55.70 ± 14.53	0.485
CD3+TCR γδ+	7.07 ± 6.42	6.91 ± 5.74	5.65 ± 5.78	0.395
CD3+CD57+	24.88 ± 11.60	26.48 ± 11.09	30.61 ± 16.70*	0.090
CD8+CD57+	43.58 ± 16.54	47.54 ± 15.07	47.24 ± 17.78	0.455
CD4+CD57+	7.43 ± 4.78	8.03 ± 6.47	13.67 ± 13.25*^Δ^	0.001
CD3+PD-1+	37.89 ± 7.50	45.30 ± 11.02^#^	54.24 ± 12.31*^Δ^	< 0.0001
CD8+PD-1+	34.79 ± 11.57	35.81 ± 11.86	43.84 ± 16.33*^Δ^	0.001
CD4+PD-1+	36.98 ± 8.24	37.84 ± 9.78	47.13 ± 16.68*^Δ^	< 0.0001
CD8+CD27+CD28+	48.10 ± 17.64	44.92 ± 14.96	42.41 ± 18.20	0.246
CD8+CD27+CD28-	7.35 ± 3.26	8.17 ± 5.39	9.02 ± 5.36	0.216
CD8+CD27-CD28+	4.13 ± 2.22	6.18 ± 5.44^#^	4.90 ± 3.24	0.042
CD8+CD27-CD28-	40.42 ± 16.62	40.73 ± 15.20	43.66 ± 18.50	0.548
CD4+CD27+CD28+	84.70 ± 7.21	84.50 ± 8.23	78.52 ± 16.14*^Δ^	0.011
CD4+CD27+CD28-	0.12 ± 0.29	0.09 ± 0.07	0.14 ± 0.13	0.401
CD4+CD27-CD28+	9.15 ± 4.72	8.95 ± 3.92	9.83 ± 6.11	0.652
CD4+CD27-CD28-	6.02 ± 4.19	6.46 ± 6.03	11.52 ± 12.48*^Δ^	0.003
CD8+ Effector T	33.44 ± 15.60	33.31 ± 16.20	31.65 ± 17.79	0.880
CD8+ Central Memory T	16.26 ± 8.98	19.31 ± 8.81	14.48 ± 9.43^Δ^	0.029
CD8+ Naïve T	17.17 ± 11.88	10.45 ± 7.95^#^	12.59 ± 12.54*	0.019
CD8+ Effector Memory T	34.13 ± 13.34	36.93 ± 12.84	41.29 ± 16.18*	0.040
CD4+ Effector T	1.22 ± 1.95	0.82 ± 0.10	1.68 ± 4.48	0.375
CD4+ Central Memory T	65.00 ± 8.34	67.90 ± 9.41	58.11 ± 15.48*^Δ^	< 0.0001
CD4+ Naïve T	17.50 ± 7.04	13.94 ± 8.82	16.59 ± 13.34	0.268
CD4+ Effector Memory T	16.27 ± 6.59	17.33 ± 8.12	23.61 ± 15.70*^Δ^	0.002
CD4+ CD39+	4.99 ± 4.00	7.78 ± 6.48^#^	8.31 ± 6.70*	0.016
CD4+CD25+FoxP3+	5.48 ± 1.70	8.55 ± 1.62^#^	9.09 ± 1.91*	< 0.0001
CD4+ CD45RA+	21.33 ± 8.40	20.43 ± 9.78	19.98 ± 13.77	0.833
CD4+ Helios+	3.74 ± 3.25	3.95 ± 3.82	3.45 ± 3.42	0.763
CD4+FoxP3+ Helios+	2.67 ± 2.63	2.85 ± 2.85	2.49 ± 2.64	0.796

Data presented as mean ± SD.

*AECOPD vs. Healthy control, *p* < 0.05,

^Δ^AECOPD vs. SCOPD, *p* < 0.05,

^#^SCOPD vs. Healthy control, *p* < 0.05. AECOPD, Acute exacerbation of chronic obstructive pulmonary disease; SCOPD, Stable chronic obstructive pulmonary disease.

### 3.3 Distribution of HLA-DR+ T-cell and TCR cell subsets

**FIGURE 3:**
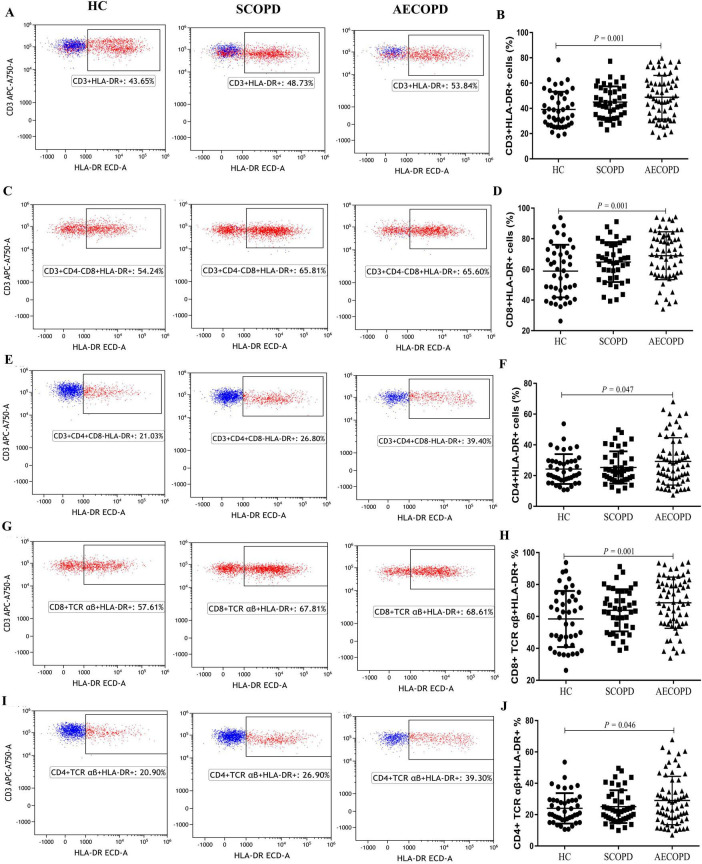
Parental proportions of HLA-DR+ T cell subsets in peripheral blood among three groups. CD3+HLA-DR+ T cells **(A,B)**, CD8+ HLA-DR+ T cells **(C,D)**, CD4+ HLA-DR+T cells **(E,F)**, CD8+TCR aβ+ HLA-DR+ T cells **(G,H)**, CD4+TCR aβ+ HLA-DR+ cells **(I,J)**. Data are expressed as mean number of each group (mean ± SD).

Compared with those in the HC group, the percentages of CD3+HLA-DR+ T cells (*P* = 0.001), CD8+HLA-DR+ T cells (*P* = 0.001), CD4+HLA-DR+ T cells (*P* = 0.047), CD8+TCR aβ+HLA-DR+ T cells (*P* = 0.001), and CD4+TCR aβ+HLA-DR+ T cells (*P* = 0.046) in the AECOPD group were significantly higher. However, although the distribution of these subsets was higher in the AECOPD group than in the SCOPD group, the difference was not statistically significant (Table 3 and Figure 3). There were also no significant differences in the proportions of CD3+TCR aβ+ T cells, CD3+TCR γδ+ T cells, CD8+TCR aβ+ T cells, or CD4+TCR aβ+ T cells among the three groups (Supplementary Figure 5).

### 3.4 Distribution of the CD57+ and PD1+ T-cell subsets

**FIGURE 4:**
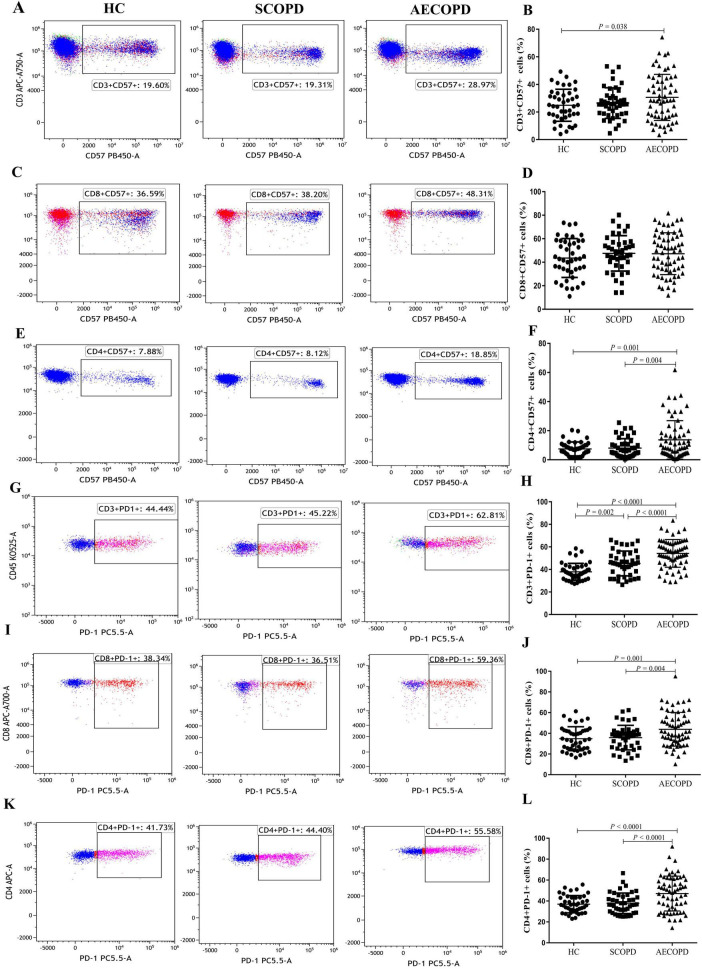
Parental proportions of CD57+ and PD1+ T cell subsets in peripheral blood among three groups. CD3+ CD57+ T cells **(A,B)**, CD8+ CD57+ T cells **(C,D)**, CD4+ CD57+ T cells **(E,F)**, CD3+ PD-1+ T cells **(G,H)**, CD8+ PD-1+ T cells **(I,J)**, CD4+ PD-1+ T cells **(K,L)**. Data are expressed as mean number of each group (mean ± SD).

The distributions of the CD57+ and PD1+ T-cell subsets are shown in Table 3 and Figure 4. The proportion of CD3+CD57+ T cells in the peripheral blood was significantly higher in the AECOPD group than in the HC group (*P* = 0.038, Figures 4A, B). Furthermore, the proportion of CD4+CD57+ T cells was significantly higher in the AECOPD group than in the SCOPD and HC groups (*P* < 0.0001 and *P* < 0.0001, respectively; Figures 4E, F). Moreover, there was no significant difference in the proportion of CD8+CD57+ T cells among the three groups (Figures 4C, D).

Compared with those in the HC group, the proportions of CD3+PD-1+ T cells in the peripheral blood were significantly higher in the AECOPD and SCOPD groups (*P* < 0.0001 and *P* = 0.002, respectively; Figures 4G, H). Concurrently, the proportions were significantly higher in the AECOPD group than in the SCOPD group (*P* < 0.0001). Further analysis revealed that the proportions of CD8+PD-1+ T cells and CD4+PD-1+ T cells were higher in the AECOPD group than in the SCOPD group (*P* = 0.004 and *P* < 0.0001, respectively) and in the HC group (*P* = 0.001 and *P* < 0.0001, respectively; Figures 4I–L), but there were no significant differences between the SCOPD group and the HC group.

### 3.5 Distribution of T-cell subsets of costimulatory molecules

**FIGURE 5:**
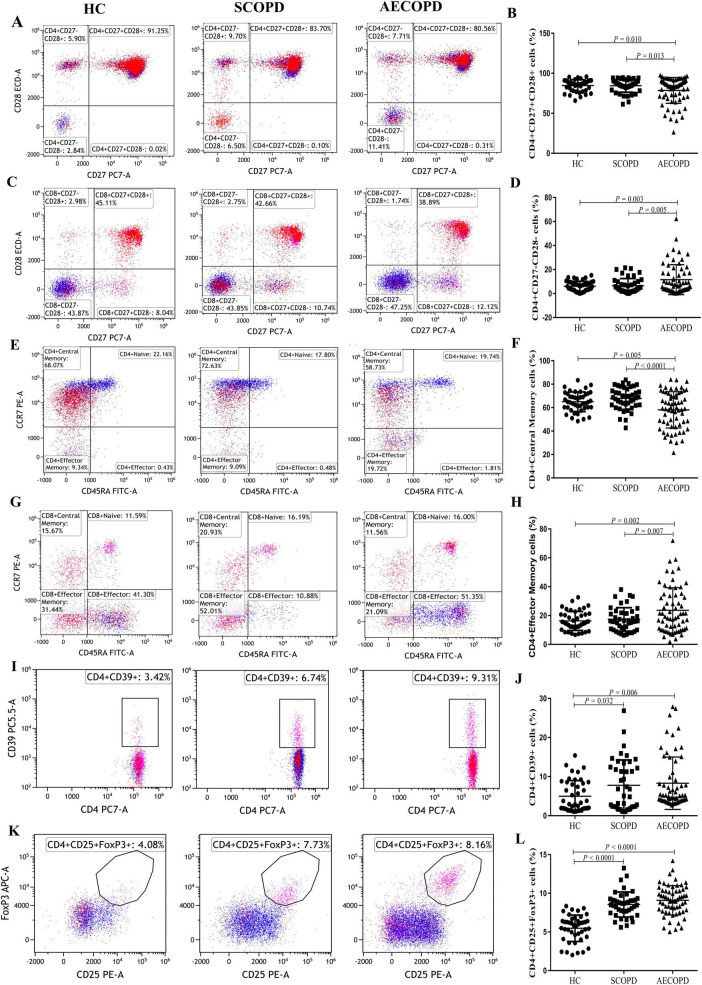
Parental proportions of antigen response T cell subsets, antigen response T cell subsets and regulatory T cell subsets in peripheral blood among three groups. CD4+CD27+CD28+ T cells **(A,B)**, CD4+CD27-CD28- T cells **(C,D)**, CD4+ Central Memory T cells **(E,F)**, CD4+ Effector Memory T cells **(G,H)**, CD4+ CD39+ T cells **(I,J)**, CD4+CD25+Foxp3+ T cells **(K,L)**. Data are expressed as mean number of each group (mean ± SD).

The distributions of the T-cell subsets associated with costimulatory molecules are shown in Table 3 and Figure 5. The proportion of CD4+CD27+CD28+ T cells was significantly lower in the AECOPD group than in the SCOPD and HC groups (*P* = 0.013 and *P* = 0.010, respectively; Figures 5A, B). In contrast, the proportion of CD4+CD27-CD28- T cells were significantly higher in the AECOPD group than in the SCOPD and HC groups (*P* = 0.005 and *P* = 0.003, respectively; Figures 5C, D). There were no significant differences in the proportions of CD8+CD27+CD28+ T cells, CD8+CD27-CD28- T cells, CD8+CD27+CD28- T cells, CD4+CD27+CD28- T cells, or CD4+CD27-CD28+ T cells among the three groups (Supplementary Figure 6).

### 3.6 Distribution of T-cell subsets in the antigen response

The distribution of T-cell subsets in the antigen response is shown in Table 3, Figure 5 and Supplementary Figure 7. The proportion of CD4+ central memory T cells was significantly lower in the AECOPD group than in the SCOPD and HC groups (*P* < 0.0001 and *P* = 0.005, respectively; Figures 5E, F). In contrast, the proportion of CD4+ effector memory T cells was significantly higher in the AECOPD group than in the SCOPD and HC groups (*P* = 0.007 and *P* = 0.002, respectively; Figures 5G, H).

### 3.7 Distribution of regulatory T-cell subsets

The distribution of regulatory T-cell subsets is detailed in Table 3 and Figure 5. The proportion of CD4+CD39+ T cells was significantly higher in the AECOPD and SCOPD groups than in the HC group (*P* = 0.006 and *P* = 0.032, Figures 5I, J), while there was no significant difference between the AECOPD and SCOPD groups. Similarly, the proportion of CD4+CD25+FoxP3+ T cells was significantly higher in the AECOPD and SCOPD groups than in the HC group (*P* < 0.0001 and *P* < 0.0001, Figures 5K, L).

### 3.8 Correlation analyses

**FIGURE 6:**
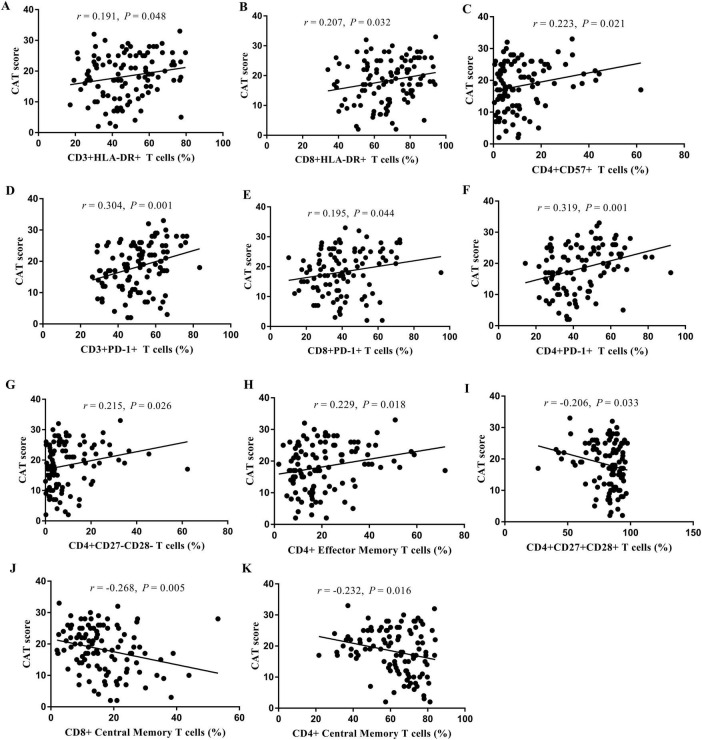
Analysis of the correlation between the proportion of T cell subsets in peripheral blood and CAT score. CD3+HLA-DR+ T cells **(A)**, CD8+HLA-DR+ T cells **(B)**, CD4+CD57+ T cells **(C)**, CD3+PD-1+ T cells **(D)**, CD8+PD-1+ T cells **(E)**, CD4+PD-1+ T cells **(F)**, CD4+CD27-CD28- T cells **(G)**, CD4+ Effector Memory T cells **(H)**, CD4+CD27+CD28+ T cells **(I)**, CD8+ Central Memory T cells **(J)**, and CD4+ Central Memory T cells **(K)**.

The results of the correlation analyses of the COPD patients are shown in Figure 6 and Supplementary Table 3. The CAT score was positively correlated with the proportions of CD3+HLA-DR+ T cells (*r* = 0.191, *P* = 0.048; Figure 6A), CD8+HLA-DR+ T cells (*r* = 0.207, *P* = 0.032; Figure 6B), CD4+CD57+ T cells (*r* = 0.223, *P* = 0.021; Figure 6C), CD3+PD-1+ T cells (*r* = 0.304, *P* = 0.001; Figure 6D), CD8+PD-1+ T cells (*r* = 0.195, *P* = 0.044; Figure 6E), CD4+PD-1+ T cells (*r* = 0.319, *P* = 0.001; Figure 6F), CD4+CD27-CD28- T cells (*r* = 0.215, *P* = 0.005; Figure 6G), and CD4+ effector memory T cells (*r* = 0.229, *P* = 0.018; Figure 6H). In contrast, the CAT score was negatively correlated with the proportions of CD4+CD27+CD28+ T cells (*r* = −0.206, *P* = 0.033; Figure 6I), CD8+ central memory T cells (*r* = −0.268, *P* = 0.005; Figure 6J), and CD4+ central memory T cells (*r* = −0.232, *P* = 0.016; Figure 6K). The correlations between the proportions of T-cell subsets and the mMRC score, FVC, and FEV_1_% pred are presented in Supplementary Table 3.

## 4 Discussion

The distribution and functional status of T-cell subsets in the peripheral blood at different phases of COPD have yet to be clarified. This study revealed that the distributions of nearly half of the T-cell subsets in AECOPD patients were significantly different from those in SCOPD patients and HCs. This study comprehensively detected the distribution and functional status of T-cell subsets in the peripheral blood of AECOPD and SCOPD patients and compared them with those in HCs using multicolor flow cytometry.

T cells mainly function as mediators of the cellular immune response (13). We found that compared with the HCs, the AECOPD patients had a significantly higher proportion of CD8+ T cells and a significantly lower proportion of CD4+ T cells. This finding is consistent with the results of previous studies and indicates that cellular immune function is suppressed (14, 15). Furthermore, we found that the number and distribution ratio of peripheral blood T cells in AECOPD patients were significantly different from those in HCs and that there was obvious inhibition of cellular immune function. However, this was not observed in patients with SCOPD, indicating that immune abnormalities differ according to the phase of COPD.

The first stage of T-cell immune function is the activation of T cells. HLA-DR is a marker of T-cell activation, but few studies have focused on HLA-DR and COPD (16, 17). The present study revealed that HLA-DR expression in CD3+, CD4+, and CD8+ T cells was significantly higher in the AECOPD group than in the HC group. However, there was no significant difference between the SCOPD group and the HC group, consistent with the findings of Pons et al. (18), who reported no significant difference in HLA-DR expression in the peripheral blood between SCOPD patients and healthy nonsmokers. In contrast, Ying et al. (19) reported significantly higher CD4+HLA-DR expression in patients with SCOPD than in normal controls and smokers. However, neither of the above two studies included AECOPD patients. Khalaf et al. (20) found that HLA-DR expression was upregulated after the alveolar macrophages of COPD patients were infected with *Haemophilus influenzae*. In summary, the distribution of HLA-DR-labeled T cells in stable COPD patients is still controversial, and the distribution of T cells in AECOPD patients has not been reported previously. Our findings suggest that there are more activated T cells in the peripheral blood of AECOPD patients than in that of HCs. The abnormal activation of T cells may be related to acute aggravating factors (such as infection) in AECOPD patients.

CD57+ T cells are very minimally expressed in the peripheral blood of newborns; however, CD57+ T cells are upregulated in chronic infections and elderly patients (21–24). Therefore, CD57+ T cells in the peripheral blood are usually regarded as terminally differentiated or senescent T cells. Olloquequi et al. (25) reported a significantly higher density of CD57+ cells in pulmonary lymphoid follicles in COPD patients than in healthy nonsmokers and smokers. Compared with moderate COPD patients, extremely patients with severe COPD have a significantly higher density of CD57+ cells in the small airways (26). The status of CD57+ T cells in the peripheral blood of patients with AECOPD has not been reported to date. As such, our finding that the proportion of CD57+ T cells in the peripheral blood was higher in the AECOPD group than in the SCOPD and HC groups is important. This finding indicates that there is an increase in T-cell senescence in AECOPD patients, further supporting the obvious inhibition of cellular immune function in AECOPD patients.

PD-1 is an important member of the CD28 family. Previous studies have shown that PD-1 expression in CD4+ T cells (27, 28) and CD8+ T (28) cells in the peripheral blood is higher in SCOPD patients than in healthy individuals. Contrasting results were observed in the current study, which could be due to differences in sample size or methods of sample treatment. However, few studies have focused on PD-1 in AECOPD patients. Only Tan et al. (29) reported that PD-1 expression in peripheral blood CD4+ T cells is increased in AECOPD patients. Consistently, the current study revealed upregulated PD-1 expression in CD3+ and CD8+ T cells in AECOPD patients. Biton et al. (30) studied the effect of COPD on non-small cell lung cancer and revealed upregulated PD-1 expression in the tumor-infiltrating CD8+ T cells of lung cancer patients with COPD. McKendry et al. (31) reported higher PD-1 expression in CD4+ T cells and CD8+ T cells in the lung tissue of COPD patients than in that of HCs. Collectively, these findings suggest that PD-1 expression is upregulated during a persistent inflammatory immune response. PD-1 is a negative regulatory costimulatory factor on effector T cells that mediates T-cell apoptosis by binding to its ligands and plays an important role in cellular immunosuppression and immune tolerance (30, 32). Therefore, PD-1 expression in peripheral T cells is increased in AECOPD patients, suggesting that more effector T cells may undergo apoptosis and reversible failure in AECOPD patients. Importantly, these findings indicate immunosuppression in AECOPD patients.

As the second signal, the costimulatory molecules CD27 and CD28 play important roles in the initial complete activation of T cells (33). They are also targets of immunosuppressive therapy. Our results showed that CD27+CD28+ (double-positive costimulatory molecule) CD4+ T cells were decreased, while CD27-CD28- (double-negative) CD4+ T cells were increased in the peripheral blood of AECOPD patients. Some studies have shown that the proliferation of highly differentiated CD28- T cells related to immune aging is associated with increased immunosuppression (34). Therefore, the increase in CD4+CD27-CD28- T cells further supports the view that there is immunosuppression in AECOPD patients.

CD39 is an extracellular nucleotidase that hydrolyzes extracellular ATP and ADP into adenosine monophosphate (AMP) and CD73 and converts AMP into adenosine. Tan et al. (35) reported that CD39 expression in CD4+, CD8+, FoxP3+, and FoxP3- T cells in the peripheral blood is increased in AECOPD patients. Consistent results were obtained in the present study. We also found that the proportion of CD4+CD25+FoxP3+ T cells (Tregs) in the peripheral blood is increased in patients with AECOPD and SCOPD. Previous studies have shown that CD39 is highly expressed in Treg cells and is important for their immunosuppressive function (36, 37). High CD39 expression is associated with other markers of T-cell depletion or dysfunction, including high PD-1 expression, low CD28 expression, and IFN-γ production (38). CD39 may promote immune failure in patients with COPD and inhibit a protective immune response.

### 4.1 Limitations

This study has several limitations. First, this was a cross-sectional study and can thus only establish the relationship between AECOPD and immune indicators and cannot suggest causality. However, this study provides the basis for further prospective follow-up studies. Second, due to the inclusion of the HC group, it was ethically impossible to further obtain lung tissue samples to understand the status of immune cells in the airway and lungs. Overall, compared with SCOPD patients, AECOPD patients had more acute exacerbations and acute hospitalizations over the past year. They also had more severe symptoms, as indicated by higher mMRC and CAT scores. It is speculated that the acute exacerbation and stable phase may also be related to the severity of the disease, and this needs to be confirmed in further subgroup studies with larger sample sizes.

## 5 Conclusion

The distributions of nearly half of the T-cell subsets in the AECOPD patients were significantly different from those in the SCOPD patients and HCs. Among patients with AECOPD, the total number of lymphocytes, the percentage of lymphocytes, and the CD4+/CD8+ T-cell ratio in the peripheral blood were significantly lower, while the proportions of negative regulatory cells (CD4+CD27-CD28- T cells, CD4+CD39+ T cells, and CD4+CD25+FoxP3+ T cells) were significantly higher, suggesting cellular immune suppression and immune dysfunction in AECOPD patients. In addition, T-cell expression of HLA-DR, CD57, and PD-1 was significantly upregulated in patients with AECOPD, indicating that there may be abnormal activation and increased senescence depletion of T cells in AECOPD.

## Data Availability

The original contributions presented in this study are included in this article/Supplementary material, further inquiries can be directed to the corresponding author.
