# Supplementary material for: T-cell immune status in patients with acute exacerbation of chronic obstructive pulmonary disease: a case-control study
Source: Front Med (Lausanne). 2025 Jan 24;12:1433844. doi: 10.3389/fmed.2025.1433844 (PMC11802415; doi:10.3389/fmed.2025.1433844)
Supplement: Supplementary file 1 [file Data_Sheet_1.pdf]

Supplemental Material

Table S1. Overview of staining panels each dedicated to a specific cell type which is indicated by individual fluorochrome

Excitation (nm)	Blue: 488					Red: 633			Violet:405	
Emission (nm)	523	575	613	692	760	650	720	767	455	528
Fluorochrome	FITC	PE	ECD	PE-Cy5.5	PE-Cy7	APC	AF700	APC-A750	PB	KRO
T cell tube	CD45RA	CCR7	CD28	PD1	CD27	CD4	CD8	CD3	CD57	CD45
T cell tube compensate	CD4	CD4	CD28	PD1	CD27	CD4	CD8	CD3	CD4	CD8
TCR tube	TCR γδ	TCR aβ	HLA-DR	-	TCR Vδ1	CD4	CD8	CD3	TCR Vδ2	CD45
TCR tube compensate	CD4	CD4	HLA-DR	-	TCR Vδ1	CD4	CD8	CD3	CD4	CD8
Treg tube	CD45RA	CD25	-	CD39	CD4	FoxP3	-	CD3	Helios	CD45
Treg tube compensate	CD4	CD4	-	CD39	CD4	FoxP3	-	CD3	CD4	CD8

Note: FITC, Fluorescein isothiocyanate; PE, Phycoerythrin; ECD, Phycoerythrin-Texas Red-X; PE-Cy, Phycoerythrin-Cyanine; APC, Allophycocyanin; AF700, Alexa Flour 700; APC-A750, Allophycocyanin Alexa Flour 750; PB, Pacific Blue; KRO, Krome Orange.

Steps for compensation setup include the following aspects:

(1) Selection and setup of compensation tubes: As detailed in Table S1, each staining panel's commercial reagent provided Duraclone single-stained tubes containing the corresponding antibodies. Additionally, we chose VersaComp Ab capture beads to substitute cells, incubating them with the Duraclone single-stained tubes containing the antibodies. Specifically, we used one drop of positive and negative VersaComp Ab capture beads for each Duraclone single-stained tube, adjusted the staining volume to 100 μl with PBS, incubated at room temperature for 15 minutes, then washed with 2 ml PBS, centrifuged at 400g for 5 minutes, removed the

supernatant, and added 500 µl PBS to resuspend the microsphere sample before running on the machine.

(2) Compensation process: We utilized the automatic compensation calculation function of the DxFLEX flow cytometer for compensation calculations. The gain for each fluorescence channel was set using QC gain to achieve excellent fluorescence resolution, and each single-stained tube collected at least 5000 microsphere particles. After the automatic compensation program finished, we acquired 10,000 lymphocytes in the sample tube and used the NxN function (pairing all fluorescence channels to form different scatter plots) to check if the compensation for all scatter plots was appropriate, manually optimizing to correct the compensation if necessary.

(3) Optimization of compensation parameters: After establishing the experimental protocol, we used this flow cytometer to acquire data. If the engineer adjusted the instrument's optical path or performed instrument maintenance, we would readjust the compensation to ensure consistency of data across experiments.

Table S2. Clinical characteristics of COPD patients at baseline

	SCOPD	AECOPD	*P*
n	43	64	
Duration of COPD, years¥	3.5(2, 6)	5.5(3.5, 10)	0.001
Cor pulmonale, n (%)	1	28	<0.001
Home oxygen therapy, n (%)	5	46	<0.001
Exacerbation history¥	0(0,1)	2(2, 3)	<0.001
Hospitalizations¥	0(0,1)	2(1, 3)	<0.001
Complications, n (%)			
Cardiovascular disease	0(0)	8(12.5)	0.014
Hypertension	9(20.9)	23(35.9)	0.132
Diabetes	1(2.3)	10(15.6)	0.047
mMRC dyspnoea score, n (%)			
0	8(18.6)	0(0)	<0.001
1	15(34.9)	0(0)	
2	20(46.5)	9(14.1)	
3	0(0)	25(39)	
4	0(0)	30(46.9)	
CAT score	11.81±5.28	22.61±4.75	< 0.001
6- minute walking distance	466.56±111.67	160.78±119.49	< 0.001
Using inhaled drugs n (%)			
LAMA	26(60.5)	37(57.8)	0.843
LABA/ICS	25(58.1)	39(60.9)	0.842
LAMA+LABA/ICS	16(37.2)	29(45.3)	0.431

Data presented as mean ± SD unless specified. ¥median (interquartile range) Pack years smoked, cigarettes per day × smoking years.

AECOPD, acute exacerbation of chronic obstructive pulmonary disease; SCOPD, stable chronic obstructive pulmonary disease; LABA, long-acting beta2-agonist; LAMA, long-acting muscarinic antagonist; ICS, inhaled corticosteroids.

Table S3. Analysis of the correlation between the proportion of T cell subsets in peripheral blood and clinical indexes

T cell subsets	CAT score		mMRC score#		FVC(L)		FEV1% pred	
	r	*P*	r	*P*	r	*P*	r	*P*
CD3+ (%)	0.026	0.787	0.085	0.382	0.128	0.374	0.181	0.190
CD3+CD8+ (%)	0.165	0.090	0.160	0.099	-0.276	0.052	**-0.286**	**0.036**
CD3+CD4+ (%)	-0.153	0.116	-0.121	0.216	0.121	0.404	0.140	0.313
CD3+HLA-DR+	**0.191**	**0.048**	0.204	0.035	-0.220	0.125	-0.102	0.464
CD8+HLA-DR+	**0.207**	**0.032**	0.185	0.056	-0.249	0.082	-0.073	0.599
CD4+HLA-DR+	0.177	0.068	**0.195**	**0.044**	-0.225	0.115	-0.108	0.438
CD8+TCR aβ+ HLA-DR+	0.213	0.027	**0.196**	**0.044**	-0.249	0.081	-0.055	0.693
CD4+TCR aβ+ HLA-DR+	0.178	0.067	**0.201**	**0.038**	-0.226	0.115	-0.106	0.443
CD3+TCR aβ+	0.147	0.131	0.145	0.136	**-0.507**	**<0.001**	**-0.362**	**0.007**
CD8+TCR aβ+	0.123	0.205	0.151	0.119	-0.211	0.142	-0.184	0.182
CD4+TCR aβ+	-0.150	0.124	-0.175	0.072	0.227	0.113	0.177	0.201
CD3+TCR γδ+	-0.147	0.130	-0.144	0.139	**0.509**	**<0.001**	**0.367**	**0.006**
CD3+CD57+	0.133	0.171	0.178	0.067	0.002	0.987	0.073	0.602
CD8+CD57+	-0.026	0.794	0.006	0.948	0.141	0.330	0.253	0.065
**CD4+CD57+**	**0.223**	**0.021**	**0.270**	**0.005**	-0.180	0.211	-0.107	0.443
**CD3+PD-1+**	**0.304**	**0.001**	**0.362**	**<0.001**	**-0.354**	**0.012**	-0.248	0.071
**CD8+PD-1+**	**0.195**	**0.044**	**0.235**	**0.015**	-0.075	0.603	-0.021	0.878
**CD4+PD-1+**	**0.319**	**0.001**	**0.369**	**<0.001**	-0.285	0.045	-0.073	0.599
CD8+CD27+CD28+	-0.146	0.134	-0.111	0.255	-0.024	0.870	-0.129	0.351
CD8+CD27+CD28-	0.076	0.435	**0.195**	**0.044**	-0.067	0.645	0.098	0.480
CD8+CD27-CD28+	-0.111	0.254	-0.080	0.415	-0.111	0.445	-0.270	0.048
CD8+CD27-CD28-	0.147	0.130	0.074	0.448	0.085	0.558	0.181	0.189
**CD4+CD27+CD28+**	**-0.206**	**0.033**	**-0.235**	**0.015**	0.263	0.065	0.183	0.186
CD4+CD27+CD28-	0.166	0.087	**0.198**	**0.041**	0.016	0.910	0.117	0.398
CD4+CD27-CD28+	0.100	0.306	0.096	0.327	**-0.378**	**0.007**	**-0.345**	**0.011**
**CD4+CD27-CD28-**	**0.215**	**0.026**	**0.276**	**0.004**	-0.138	0.339	-0.054	0.700
CD8+ Effector T	-0.036	0.710	-0.071	0.467	0.068	0.638	0.195	0.157
**CD8+ Central Memory T**	**-0.268**	**0.005**	**-0.303**	**0.002**	0.005	0.970	-0.129	0.354
CD8+ Naïve T	0.010	0.915	-0.075	0.441	0.022	0.880	-0.098	0.483
CD8+ Effector Memory T	0.202	0.037	**0.259**	**0.007**	-0.102	0.481	-0.090	0.516

CD4+ Effector T	0.053	0.587	0.107	0.273	-0.047	0.748	0.075	0.589
**CD4+ Central Memory T**	**-0.232**	**0.016**	**-0.280**	**0.004**	0.107	0.459	0.035	0.802
CD4+ Naïve T	0.001	0.989	-0.077	0.433	0.128	0.376	0.106	0.445
**CD4+ Effector Memory T**	**0.229**	**0.018**	**0.296**	**0.002**	-0.259	0.069	-0.159	0.251
CD4+ CD39+	0.014	0.884	**0.220**	**0.025**	0.067	0.647	0.136	0.331
CD4+CD25+FoxP3+	0.090	0.362	0.184	0.061	-0.009	0.951	-0.199	0.153
CD4+ CD45RA+	-0.073	0.459	**-0.210**	**0.032**	0.235	0.103	0.183	0.189
CD4+ Helios+	-0.027	0.784	-0.070	0.478	-0.240	0.096	-0.099	0.482
CD4+FoxP3+ Helios+	-0.031	0.758	-0.116	0.242	**-0.332**	**0.020**	-0.170	0.223

#Spearman correlation test.

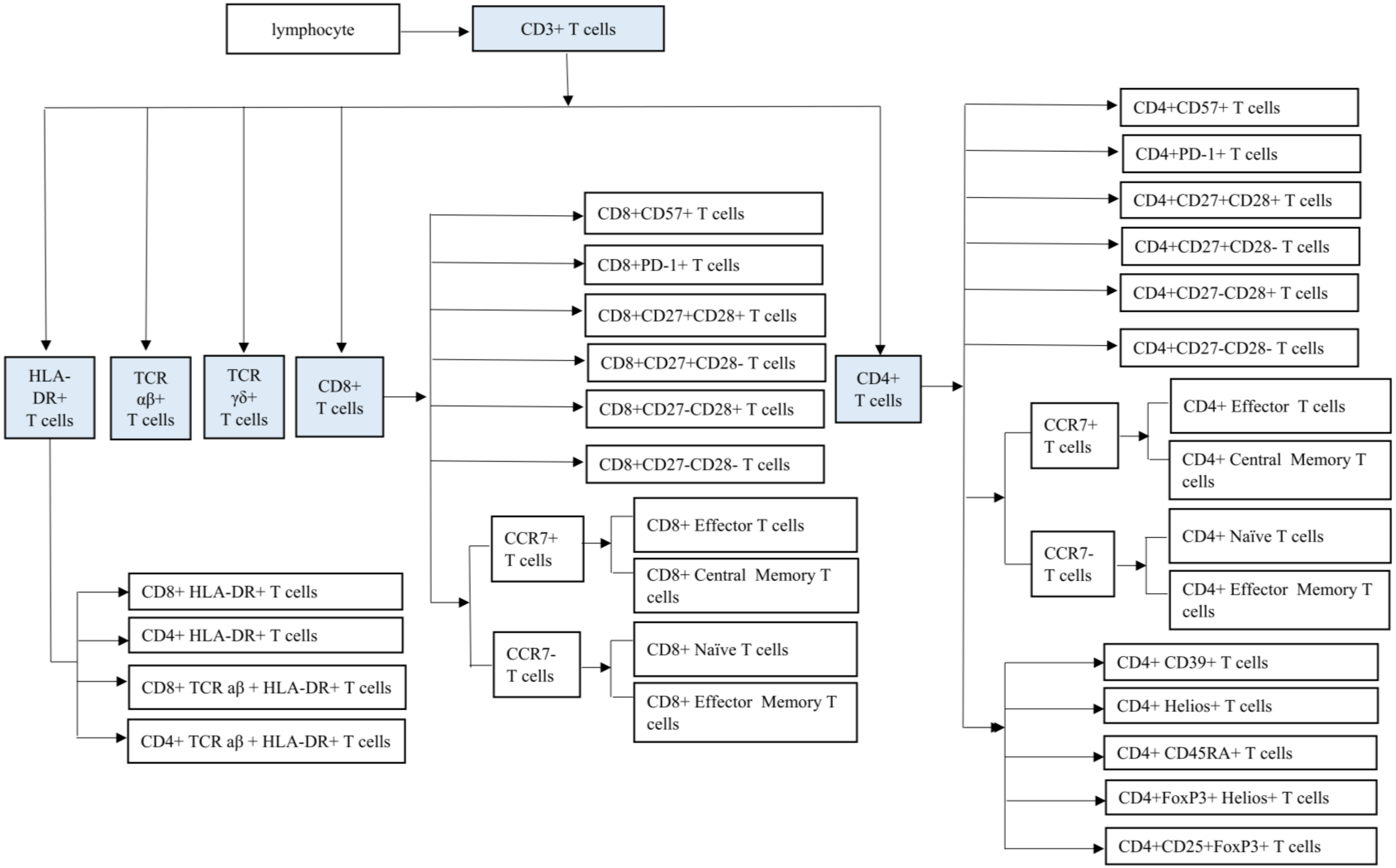

Figure S1. Schematic overview and flow cytometric gating strategies of T cell subsets in peripheral blood

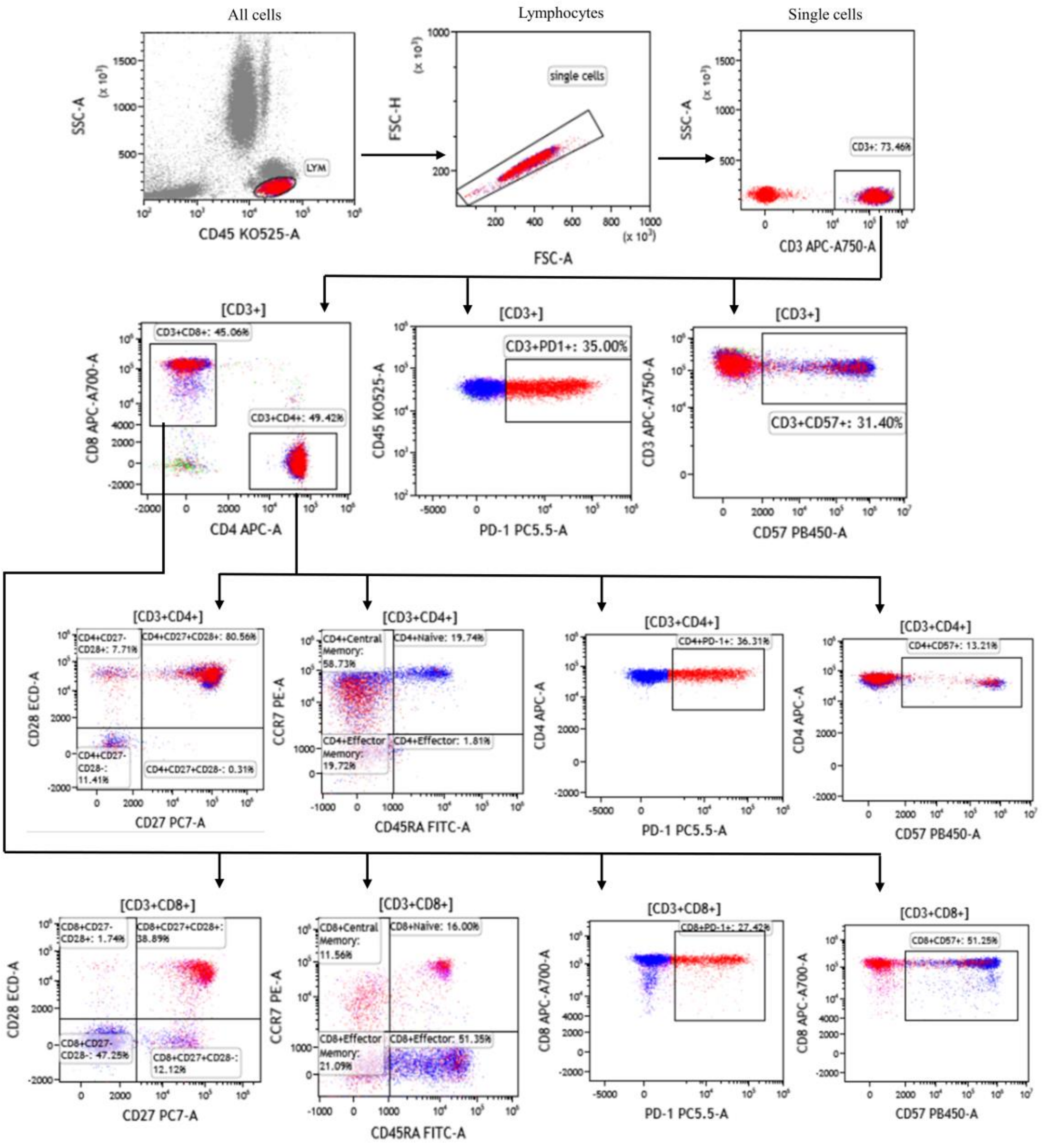

Figure S2. Gating strategy for T cell tube. Lymphocytes were gated based on high expression of CD45 and low side scatter (SSC), and a single lymphocyte is gated by the combination of A and H signals of forward scatter (FSC), named by single cells. Lymphocytes were then classified based on CD3 expression to identify T cells, which were divided into CD4+ and CD8+ T cells, prior to separation into their four main subsets CD27+CD28+, CD27+CD28-, CD27-CD28+, and CD27-CD28- T cells, this separation was based on the expression of CD27and CD28; At the same time, according to the expression of CCR7 and CD45RA, CD4+ and CD8+ T cells were divided into central memory, naïve, effector and effector memory T cells. The CD3+, CD4+ and CD8+ T cells were subsequently analysed for expression of the markers PD-1 and CD57.

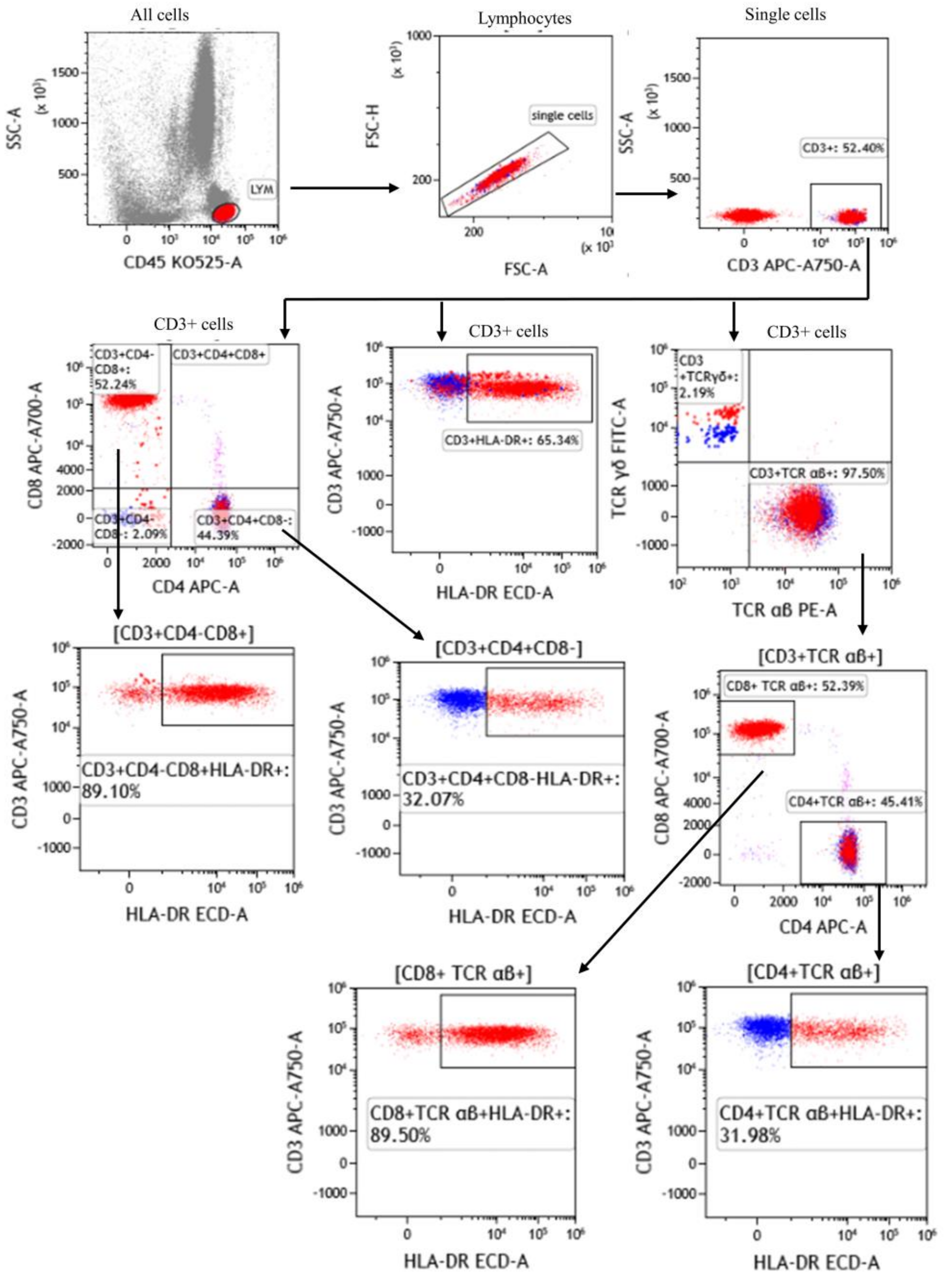

Figure S3. Gating strategy for TCR tube. Lymphocytes were gated based on high expression of CD45 and low side scatter (SSC), and a single lymphocyte is gated by the combination of A and H signals of forward scatter (FSC), named by single cells. Lymphocytes were then classified based on CD3 expression to identify T cells, which were divided into CD4+ and CD8+ T cells; at the same time, CD3+T cells were divided into CD3+TCR αβ+ T cells and CD3+TCR γδ+ T cells based on the expression of TCR αβ and TCR γδ, then, CD3+TCR αβ+ T cells were divided into CD4+ and CD8+ TCR αβ+ T cells. Finally, the CD3+, CD4+, CD8+, CD4+TCR αβ+ and CD8+ TCR αβ+ T cells were analysed for expression of HLA-DR.

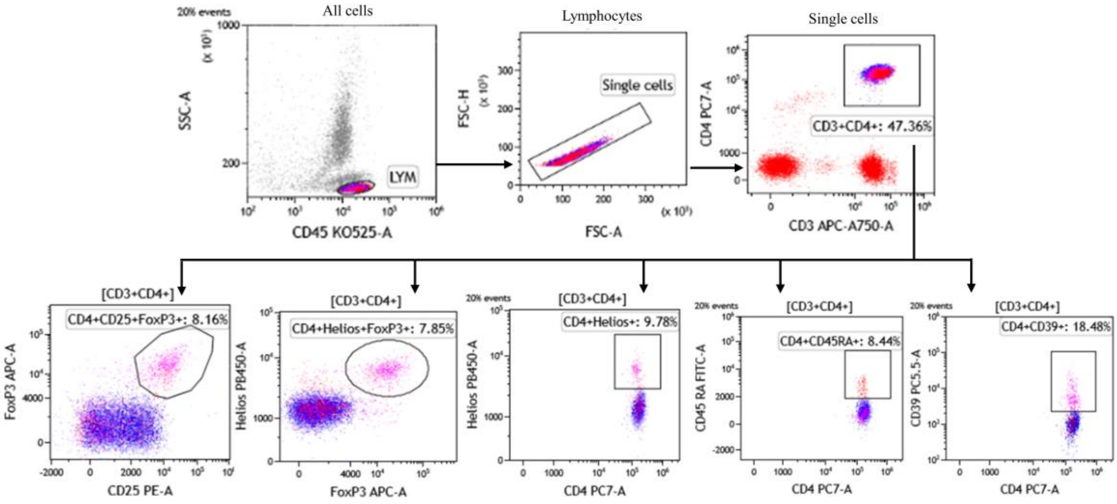

Figure S4. Gating strategy for Treg tube. Lymphocytes were gated based on high expression of CD45 and low side scatter (SSC), and a single lymphocyte is gated by the combination of A and H signals of forward scatter (FSC), named by single cells. Lymphocytes were then classified based on CD3 and CD4 expression to identify CD4+T cells, which were divided into five subsets CD4+CD39+ T cells, CD4+CD25+ Forkhead box protein 3 (FoxP3) + T cells, CD4+CD45RA+ T cells, CD4+Helios+ T cells, CD4+FoxP3+Helios+ T cells.

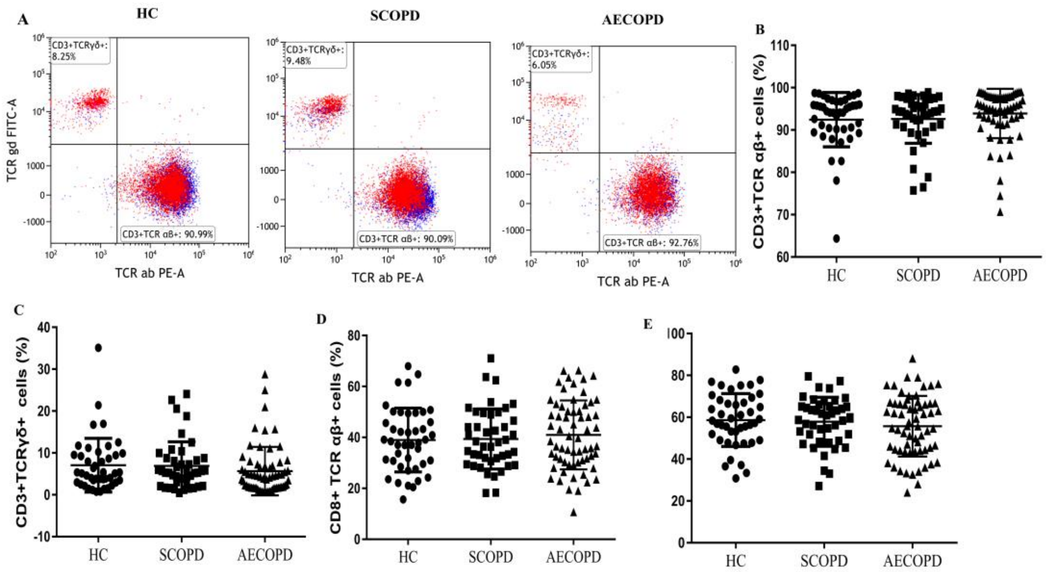

Figure S5. Parental proportions of TCR T cell subsets in peripheral blood among three groups. CD3+TCR aβ+ T cells (A, B)、CD3+TCR γδ+ T cells (C)、CD8+ TCR aβ+ T cells (D)、CD4+ TCR aβ+ T cells (E). Data are expressed as mean number of each group (mean ± SD).

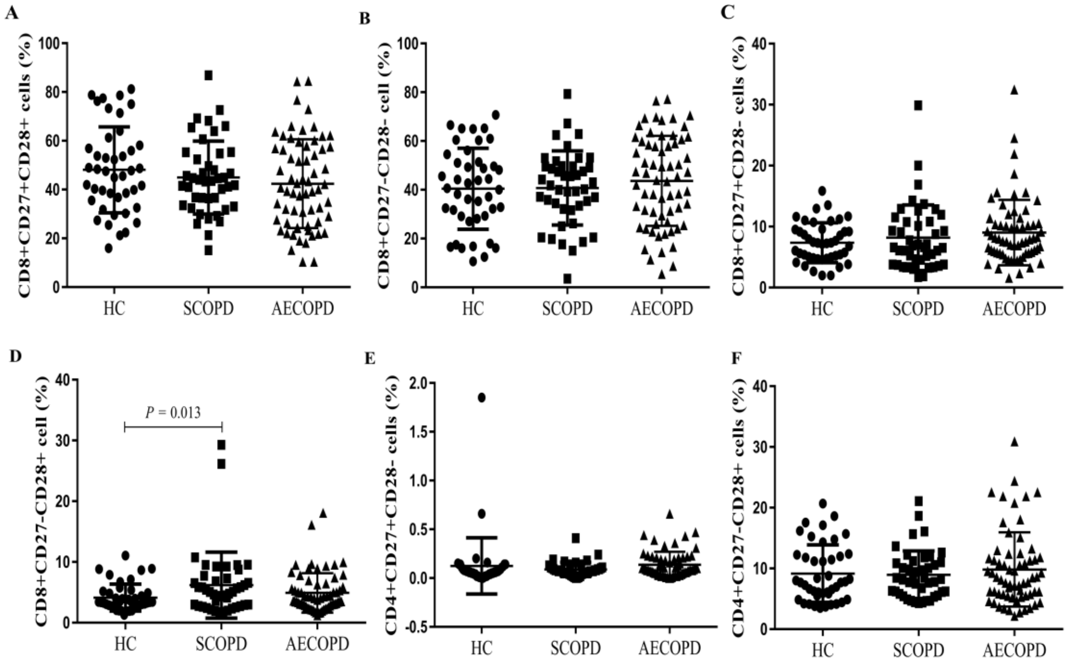

Figure S6. Parental proportions of costimulatory molecules T cell subsets in peripheral blood among three groups.

CD8+CD27+CD28+ T cells(A)、CD8+CD27-CD28- T cells(B)、CD8+CD27+CD28- T cells(C)、CD8+CD27-CD28+ T cells(D)、CD4+CD27+CD28- T cells(E)、CD4+CD27-CD28+ T cells(F). Data are expressed as mean number of each group (mean ± SD).

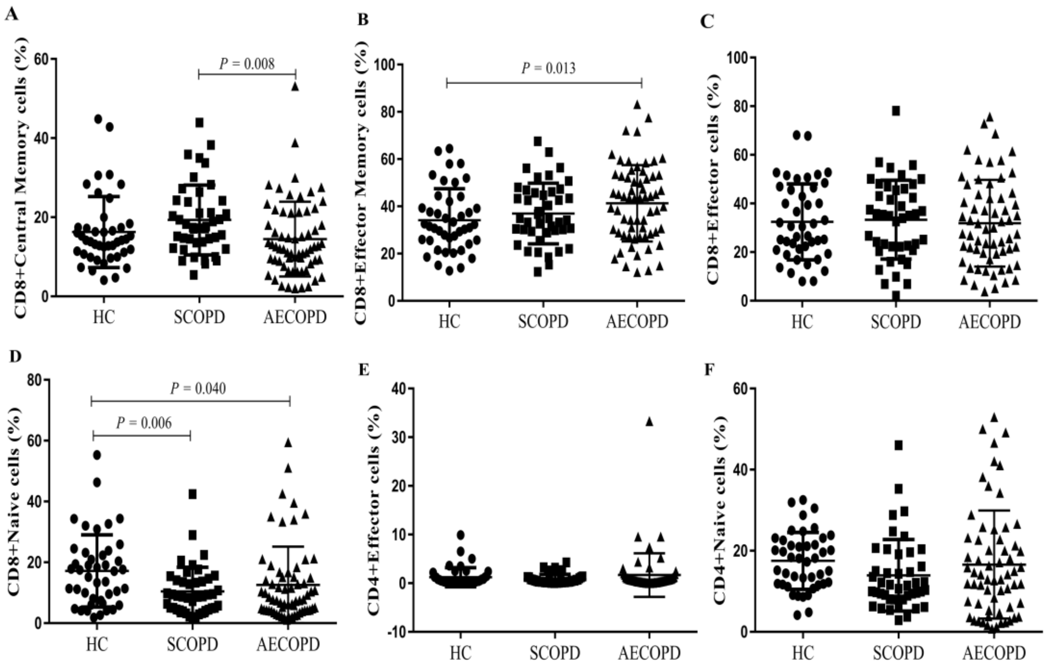

Figure S7. Parental proportions of antigen response T cell subsets in peripheral blood among three groups.CD8+ Central Memory T cells(A)、CD8+ Effector Memory T cells(B)、CD8+ Effector T cells(C)、CD8+ Naïve T cells(D)、CD4+ Central Memory T cells(E)、CD4+ Naïve T cells(F). Data are expressed as mean number of each group (mean ± SD).
